# Outcomes of Nipple-Sparing Mastectomy in the Ptotic and Non-Ptotic Breast with Staged-Immediate Reconstruction Timing and Pre-Pectoral, Direct-to-Implant Technique

**DOI:** 10.7759/cureus.42363

**Published:** 2023-07-24

**Authors:** Mary Duet, Ivo A Pestana

**Affiliations:** 1 Plastic and Reconstructive Surgery, Atrium Health Wake Forest Baptist Medical Center, Winston-Salem, USA

**Keywords:** breast reconstruction, direct to implant technique, comparative study, patient outcomes, breast ptosis, breast reconstruction timing, prepectoral breast reconstruction, implant breast reconstruction, nipple-sparing mastectomy

## Abstract

Background and objectives

Proven to be oncologically safe, nipple-sparing mastectomy (NSM) preserves the entire breast skin envelope and is associated with higher patient satisfaction. However, breast ptosis is a relative contraindication to NSM, limiting who it is offered to. Direct-to-implant (DTI) breast reconstruction eliminates tissue expansion and shortens the reconstructive process but may be associated with mastectomy skin flap compromise after the placement of full-volume implants. Staged-immediate (SI) reconstruction initiates reconstruction two to three weeks after mastectomy. This timing and its use in DTI pre-pectoral (PP) breast reconstruction have not been reported. We aim to describe the outcomes of SI DTI PP reconstruction following NSM of ptotic and non-ptotic breasts.

Methods

Retrospective analysis utilizing descriptive statistics was completed evaluating patients who underwent nipple-sparing mastectomy with staged-immediate, pre-pectoral, direct-to-implant reconstruction by the senior author over a three-year period.

Results and conclusions

With SI timing, the majority of mastectomy-related problems occurred prior to implant placement, likely mitigating their effects on reconstruction following NSM, regardless of ptosis grade. Although a second procedure is needed for this reconstructive timing variation, over 50% of women achieved reconstruction completion at implant placement without further revision. These findings support the utility of SI timing in PP DTI reconstruction following NSM.

## Introduction

The surgical treatment and prevention of breast cancer, one of the most common malignancies in the United States, are rapidly evolving. Proven to be oncologically safe, nipple-sparing mastectomy (NSM) preserves the entire breast skin envelope. Nipple-sparing mastectomy is associated with higher patient satisfaction, improved body image, and psychological adaptation following mastectomy, as well as nipple sensitivity, when compared to skin-sparing mastectomy (SSM) [1]. In addition, NSM incision patterns and their use tend to change over time, contributing to improved cosmetic outcomes [2]. Unfortunately, women with breast ptosis are often not considered for NSM due to the increased risk of nipple ischemia and nipple-areolar complex (NAC) malposition [3-5]. The popularity and benefits of NSM have produced an increasing need to offer this mastectomy technique to the breast ptosis patient population.

Implant-based breast reconstruction (IBBR), the most common form of reconstruction worldwide, has dramatically improved from a patient and surgeon perspective due to advancements in devices and techniques. Currently, the most common type of implant-based reconstruction is two-staged with muscular coverage of the implant [6]. The introduction and development of acellular dermal matrices (ADMs) and other biosynthetic meshes have allowed for partial submuscular placement, which contributes to improved control of lower pole fullness and reduced tissue expansion times [7-8].

The pre-pectoral (PP) implant location has re-emerged to eliminate the drawbacks associated with submuscular implant placement. Pre-pectoral implant placement is associated with less postoperative pain and avoids the animation deformity associated with implants placed under the pectoralis major muscle [8-9]. Direct-to-implant (DTI) strategies can dramatically decrease the overall length of the reconstruction process, but the inappropriate implementation of DTI reconstruction may lead to increased morbidity due to abrupt stress being placed onto the mastectomy flap and NAC, which has been cited to have rates of nipple and skin necrosis as high as 38% [10].

Recently, modifications to reconstructive timing have been popularized. Staged-immediate (SI) reconstruction timing, also known as the Zenn delay, initiates reconstruction about two to three weeks after mastectomy. The staging of implant placement allows for the identification of skin flap perfusion problems, recovery from superficial injury, and full-thickness tissue injury to declare itself ultimately with the aim of reducing the complication rates of implant-based reconstruction following mastectomy [10].

The use of staged-immediate reconstruction timing with direct-to-implant reconstruction in the pre-pectoral plane has not been reported. Here we describe our experience and present a review of outcomes from a single surgeon's experience with staged immediate (SI), pre-pectoral (PP), direct-to-implant (DTI) reconstruction after NSM of the ptotic and non-ptotic breast.

## Materials and methods

Population analysis

An institutional review board-approved retrospective analysis was completed evaluating consecutive patients who underwent nipple-sparing mastectomy with staged-immediate, direct-to-implant reconstruction in the pre-pectoral plane by the senior author over a three-year period at the Atrium Health Wake Forest Baptist Medical Center, Winston-Salem, USA (approval number: IRB00056367). Patients were identified using the International Classification of Diseases (ICD) codes. Identified patients underwent chart review to (1) confirm that NSM with SI DTI PP implant placement was performed and (2) confirm the patient had at least one month of follow-up after placement of the final implants. Patients with skin-sparing mastectomy, two-stage implant reconstruction, submuscular implant placement, reconstruction timing other than SI, less than one month of follow-up from their final reconstructive procedure, and patients with reconstruction using autologous flaps were excluded. Data relevant to the study were extracted from patient charts. A data collection tool was created to capture information related to patient demographics, indications for surgery, technical details related to reconstruction, and outcomes.

Specific data points utilized in this analysis included the reason for mastectomy (therapeutic versus prophylactic), the incidence of complications, patient age, and comorbid conditions. In this study, a major complication was defined as those leading to implant loss, an unplanned return to the operating room, or hospital admission. Minor complications were defined as those not leading to implant loss, an unplanned return to the operating room, or hospital admission.

Descriptive statistics were calculated for demographics, patient-specific characteristics, clinical characteristics, reconstructive details, and outcomes per patient and per breast.

Technique

All patients were evaluated by the senior author preoperatively. During that encounter, the SI DTI PP reconstruction strategy was discussed. Patients were counseled regarding planned staging or the need to stage implant placement after mastectomy based on patient-related factors, such as ptosis, the need for mastopexy, and the assessment of skin flap perfusion. The incisional pattern for NSM was made at the discretion of the surgical oncologist with collaborative input from the reconstructive surgeon.

An inframammary fold (IMF) incision was most frequently performed by surgical oncologists, and concomitant axillary nodal procedures, if indicated, were performed through a separate axillary incision. If Wise pattern skin reduction was planned preoperatively, the NSM was performed via a triangular incision contained within the planned skin resection lines of the caudal breast. If vertical skin reduction was planned, NSM was performed via a longitudinally oriented elliptical incision inferior to the NAC. Sub-nipple duct samples were sent for frozen section pathology in all patients prior to the initiation of reconstruction.

Flap thickness, color, capillary refill, and dermal bleeding were assessed intra-operatively by the senior author after the completion of the mastectomy to determine the adequacy of skin flap perfusion. If clinical assessment raised concern, indocyanine green (ICG) fluorescent angiography was used as an adjunct to a physical exam in cases of planned immediate reconstruction to determine the need to stage implant placement. Patients undergoing planned SI reconstruction or those switched to staged-immediate timing underwent skin closure over drains either by the surgical oncology team or the plastic surgeon. Seven to ten days post-mastectomy, the patient was seen in the clinic, and the upcoming procedure was discussed in regards to technique and timing, which were determined by assessment of skin and NAC ischemia.

At the time of SI implant placement, the mastectomy incisions were opened and the skin flaps were elevated off the chest wall muscles. Any areas of full-thickness soft tissue injury were excised. Implants were then placed in the PP plane with acellular dermal matrix (ADM), DermACELL (LifeNet Health, Virginia Beach, Virginia), or AlloDerm (LifeCell Corp., Branchburg, New Jersey), with either implant wrap or "anterior tent" technique following implant sizer use to determine appropriate final implant volume. Concurrent breast shaping procedures at the time of implant placement were performed prior to the placement of the final implant with a temporary sizer in place. Implant pockets were irrigated with a dilute povidone-iodine solution (10%), and the final implants were only handled by the senior author. Drains (15 French, channeled) were placed in all cases and removed based on output volume trends. After implant placement, patients were discharged on oral antibiotics for seven days, with specific drugs determined based on the patient's allergy profile and our institution’s antibiogram.

## Results

**Figure 1:**
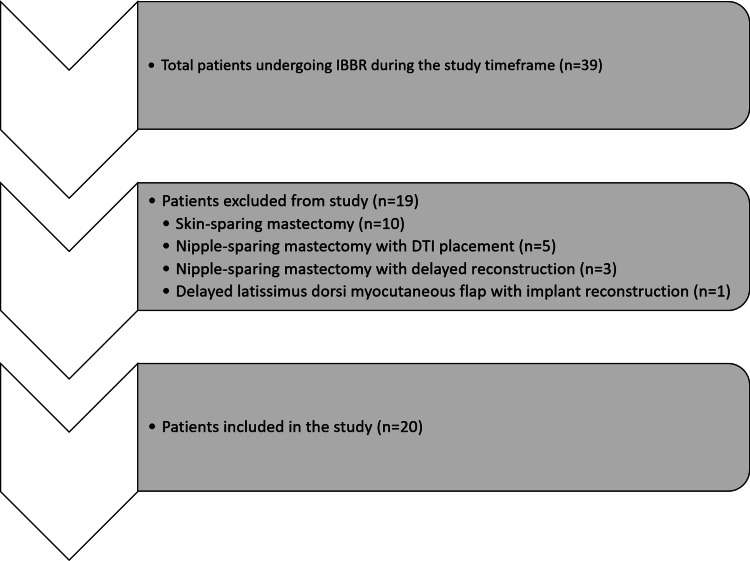
Study patient inclusion and exclusion criteria IBBR: implant-based breast reconstruction

During the study period, a total of 39 patients underwent implant-based breast reconstruction by the senior author. Nineteen patients were excluded due to surgical techniques outside the study parameters, including 10 patients who underwent skin-sparing mastectomy (SSM) and one patient who had a delayed latissimus dorsi myocutaneous flap and implant reconstruction. Eight of the 19 excluded patients underwent NSM, five of whom had immediate DTI placement, and three experienced delayed reconstruction due to tissue loss. Of the 39 considered patients, 20 women, with a total of 35 breasts, underwent SI DTI reconstruction in the PP plane after NSM and were included in the study analysis (Figure 1).

Patient characteristics are described in Table 1.

**Table 1:** Patient characteristics SD: standard deviation; BMI: body mass index; ASA: American Society of Anesthesiologists Physical Status Classification; DVT/PE: deep vein thrombosis/ pulmonary embolism

Patient characteristics	
Total patients, n	20
Number of breasts reconstructed, n	35
Age, Mean (SD), years	49.8 (12.07)
Follow-up, Mean (SD), months	25.5 (10.34)
BMI, mean (SD), Kg/ m2	24.3 (3.17)
ASA status, Mean	2.1
Neoadjuvant or adjuvant chemotherapy, n, (%)	4 (20%)
Radiotherapy, n, (%)	2 (10%
Diabetes, n, (%)	0 (0%)
History of smoking, n, (%)	5 (25%)
Active tobacco use, n, (%)	0 (0%)
History of DVT/ PE, n, (%)	1 (5%)
Hypertension, n, (%)	0 (0%)
Autoimmune diagnosis, n, (%)	0 (0%)
Hyperlipidemia, n, (%)	0 (0%)
Mental health diagnosis, n, (%)	8 (40%)

The average patient age was 49.8 years, with 70% of the patients falling between the ages of 40 and 60. The average body mass index (BMI) for this cohort was 24.3 kg/m2, with one patient meeting the World Health Organization (WHO) criteria for obesity. None of the patients actively used tobacco products or were diagnosed with diabetes mellitus. Previous tobacco use was reported by 25% of the study population. A bilateral mastectomy was performed in 75% of patients. Indications for mastectomy were largely therapeutic for cancer, with five patients undergoing the procedure for prophylaxis (25%). Patients with cancer were all stage 1 or less. Two patients received adjuvant radiation, and no oncological treatment was delayed due to the surgical techniques discussed.

An inframammary fold (IMF) incision was performed in 75% of the included patients by surgical oncologists. In one case, after completion of NSM via IMF incision, the incision pattern was converted to a skin-reducing pattern. At the time of implant placement, two patients with IMF incision patterns were converted to a skin-reducing pattern. In total, 12 patients had IMF incisions without skin reduction. At the time of the mastectomy, two patients received a skin-reducing vertical incision, while three other patients underwent Wise pattern skin reduction. One patient underwent a radial incision without skin reduction. In total, seven out of the 20 patients included (35%), all of whom had grade 2 or 3 ptosis, underwent mastectomy with skin reduction.

Indications for SI timing included breast ptosis and skin perfusion compromise after mastectomy, either determined by surgeon assessment of the skin flaps or NAC or in conjunction with ICG fluorescent angiography, which was performed in 35% of the included patients. Staged-immediate placement of the implant was performed on average 14.7 days (range 12-19 days) after mastectomy in all patients (Table 2).

**Table 2:** Breast reconstruction characteristics cc: cubic centimeter implant volume

Breast reconstruction characteristics	
Time to implant placement, Mean (SD), days	14.7 (1.69)
Patients with concurrent procedure to mastectomy, n, (%)	5 (25%)
Patients with concurrent procedure to implantation, n, (%)	6 (30%)
Implant volume, Mean (SD), cc	402.8 (126.59)
Time to reconstruction completion, Mean (SD), months	3.18 (3.44)
Time to reconstruction completion without complication, Mean (SD), months	0.91 (1.24)
Time to reconstruction completion with complication, Mean (SD), months	4.7 (3.62)

Half of the included patients had grade 2 ptosis or higher. Of the patients included, seven women planned for immediate reconstruction were switched to SI timing due to fluorescent angiography findings or clinical exam findings. As delineated above, concurrent breast shaping procedures (i.e., mastopexy variations) at the time of mastectomy were performed on five patients (25%), all of whom had a ptosis grade of 2 or higher. Six patients (30%), also with a ptosis grade of 2 or higher, underwent concurrent procedures at implant placement. Of the six patients who underwent concurrent procedures at the time of implant placement, four had undergone a mastopexy at the time of the mastectomy as well. Of these four patients, following the original mastectomy and mastopexy, two underwent contralateral mastopexies for symmetry, one underwent medialization of the NAC, and one had local tissue rearrangement performed.

The overall complication rate was 60%, comprised of 22 discrete complications in 12 patients (Table 3).

**Table 3:** Adverse event summary

Adverse event summary	Patient (n=20)	Breasts (n=35)
Experience of at least one complication, n, (%)	12 (60%)	18 (51.43%)
Discrete major complications, n, (%)	4 (20%)	4 (11.43%)
Major complication prior to implantation, n, (%)	0 (0%)	0 (0%)
Major complication after implantation, n, (%)	4 (20%)	4 (11.43%)
Discrete minor complications, n, (%)	10 (50%)	14 (40%)
Minor complication prior to implantation, n, (%)	9 (45%)	17 (48.6%)
Minor complication after implantation, n, (%)	1 (5%)	1 (2.86%)

Within those 12 patients, 18 breasts experienced complications, for a total breast complication rate of 51.4%. Major and minor complications occurred in 20% and 50% of patients, respectively. No major complications occurred prior to the placement of the implants. Major complications included two unilateral implant losses and two patients requiring hospitalization for IV antibiotics for unilateral cellulitis, in total affecting four patients and four individual breasts. Ten patients experienced 18 minor complications. Of these minor complications, 17 (94%) occurred prior to the placement of implants, and one (11%) occurred after the placement of implants (Table 3). Pre-implant minor complications consisted of superficial tissue injuries of the nipple or skin flaps that required wound care, and post-implant minor complications consisted of one patient with inframammary wounds requiring wound closure under local anesthesia. No NAC loss occurred after implant placement. Patients with grade 1 ptosis experienced a total complication rate per breast of 44%, while those with breast ptosis grade 2 or higher experienced a complication rate per breast of 55% (Table 4).

**Table 4:** Ptosis and adverse events

Ptosis and adverse events			
Grade 1 ptosis		Patients (n=10)	Breasts (n=18)
	Major complication, n, (%)	2 (20%)	2 (11%)
	Minor complication, n, (%)	5 (50%)	8 (44%)
Grade 2 and 3 ptosis		Patients (n=10)	Breasts (n=17)
	Major complication, n, (%)	2 (20%)	2 (11.8%)
	Minor complication, n, (%)	5 (50%)	6 (35.3%)

Eleven (55%) patients completed reconstruction at implant placement, and nine (45%) underwent revision with fat grafting or mastopexy following implant placement, including those who required explantation and subsequently underwent tissue expansion. Fourteen revisionary procedures were performed in the study population within one year of mastectomy, not including concurrent procedures performed at the time of mastectomy or implant placement.

For this cohort, the average number of surgeries was 2.3, with an average of 1.65 hospitalizations to complete reconstruction. In patients without complications, the average time to complete reconstruction, including revisionary procedures, was 0.91 months. In comparison, patients who experienced complications took on average 4.7 months to complete reconstruction, with revisionary procedures accounted for (Table 2).

## Discussion

The majority of breast reconstruction procedures following mastectomy involve the use of an implantable prosthesis, typically a tissue expander placed into a submuscular pocket, followed by replacement with a final implant after sufficient expansion of the skin envelope [11]. Utilization of the NSM technique preserves the skin envelope and may obviate the need for expansion while also remaining oncologically equivalent to traditional mastectomy in well-selected patients [12]. As a result, direct-to-final implant reconstruction without expansion is also becoming more common. Furthermore, pre-pectoral implant placement is gaining popularity as it avoids animation deformity, is associated with less postoperative pain, and improved cosmetic outcomes [2,13]. However, inappropriate application of this technique may result in serious complications such as implant loss and the subsequent need for unplanned or additional operative procedures.

The literature examining outcomes of patients undergoing NSM, particularly those with breast ptosis, and DTI PP reconstruction with staged-immediate reconstruction timing is limited. Furthermore, reported outcomes of DTI PP reconstruction with immediate timing vary. Jones and Anthony published a series of 140 patients who underwent immediate, direct-to-implant, pre-pectoral breast reconstruction and identified a cellulitis rate of 0%-5.7% and an implant loss rate of 0%-6.7% per patient. Mastectomy type and ptosis grade were not reported, nor were simultaneous breast-shaping procedures performed at the time of the initial operation [14]. Additionally, successful outcomes were reached in 93.3% of cases in one surgeon’s patient cohort and 100% of patients in the other surgeon’s patient group [14]. A large, multi-center prospective study performed in the United Kingdom reported short-term safety outcomes within three months of immediate implant-based breast reconstruction. Of the 2,108 patients included, 42 patients, or 2% of the study population, received immediate PP implant reconstruction with the utilization of mesh (unreported mesh type) [15]. Of those having PP plane reconstruction, 22 had NSM, 36 underwent DTI placement, and five also underwent concurrent skin reduction. Reported results of patients with PP implant placement included a readmission rate of 24%, an infection rate of 26%, and a reoperation rate of 21%, with 7% of this subgroup experiencing implant loss. The previously stated results were not further specified for those receiving PP placement with DTI or NSM. Of note, preoperative ptosis grades and intraoperative perfusion assessment techniques were not recorded or analyzed [15].

In this review of our experience with SI DTI PP reconstructions in patients with NSM, 60% of patients, or 51.4% of reconstructed breasts, experienced at least one complication. Seventeen discrete complications occurred prior to implant placement after a mastectomy, all of which were minor. Within our population, two patients with bilateral procedures (10% of the population, or 5.7% of the breasts) experienced unilateral implant loss. One implant was lost approximately three months after SI placement due to full-thickness tissue loss and implant exposure. Another patient lost an implant secondary to infection shortly after placement. Both patients have since completed a two-stage implant reconstruction with satisfactory results.

When compared to immediate DTI PP reconstruction reported in the literature, critical information regarding the technique of SI DTI PP implant-based reconstruction may be identified. First, the complication profile of SI DTI PP reconstruction following NSM in the ptotic and non-ptotic groups is similar to that of cases performed immediately at the time of other mastectomy types (SSMs). Indeed, a preliminary analysis made prior to the episode of delayed implant loss resulted in an implant loss rate of 2.8% per breast, which is somewhat lower than reported in the literature for immediate DTI PP reconstruction in appropriately selected patients [15]. These overall low rates of complications after staged reconstruction indicate that it is a safe strategy in both ptotic and non-ptotic patient populations. The proportionally larger number of complications that occurred prior to implant placement, though mostly minor in nature, indicate that staging implant placement may have reduced the number of complications and possibly the incidence of major complications, if implant placement had occurred at the time of mastectomy.

In comparison to immediate DTI reconstruction, the additional surgical procedure required by the staged timing may be considered a disadvantage since it may extend the time to reconstruction completion. However, the increased length is much shorter than the accepted delays associated with tissue expansion. Moreover, the potential to avoid complications that may lead to patient morbidity, in our opinion, may justify the extra time and additional surgical procedure. One may argue that the two- to three-week interlude between mastectomy and implant placement is advantageous for the ptotic patient. As delineated above, 50% of our population had Regnault grade 2 ptosis or higher, yet only 35% underwent skin reduction. This discrepancy may be explained by the natural contraction of breast skin in the interim between mastectomy and implant placement, obviating the need for skin reduction [16]. In addition, the ischemic preconditioning or "vascular delay" that is inherent to staged-immediate reconstruction timing may allow the use of breast implants with more volume than the native breast gland, minimizing the need for skin resection at implant placement.

**Figure 2:**
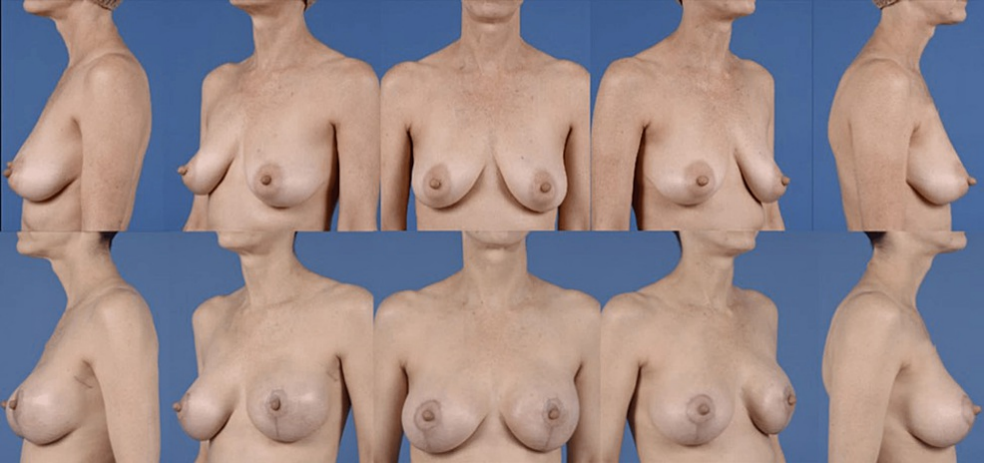
Preoperative and six-month postoperative comparison of ptotic breasts that underwent nipple-sparing mastectomy with staged-immediate, direct-to-implant, pre-pectoral implant-based breast reconstruction

Importantly, by staging the placement of an implant, patients with ptotic breasts may be able to receive an NSM, which is historically a relative contraindication for NSM due to higher rates of NAC ischemia [17]. In our series, 50% of patients had Regnault grade 2 ptosis or higher, and all achieved reconstruction completion without NAC loss (Figure 2).

One may postulate that offering and successfully completing NSM and reconstruction to patients who may not otherwise be considered for it due to their degree of ptosis may shorten the total length of reconstruction time and minimize costs associated with NAC reconstruction.

Regarding revisions of cases completed with the study timing and technique, over half of the patients achieved reconstruction completion following implant placement, on average 14.7 days following mastectomy. Revisionary procedures included 14 total procedures performed for nine patients and included common interventions employed for the improvement in breast footprint, nipple-areolar complex positioning, and breast volume (i.e., mastopexy or its revision if completed at the time of mastectomy, autologous fat transfer). Revisionary rates in the literature vary when compared to our study, likely due to differences in population size, subject characteristics, and techniques utilized.

**Figure 3:**
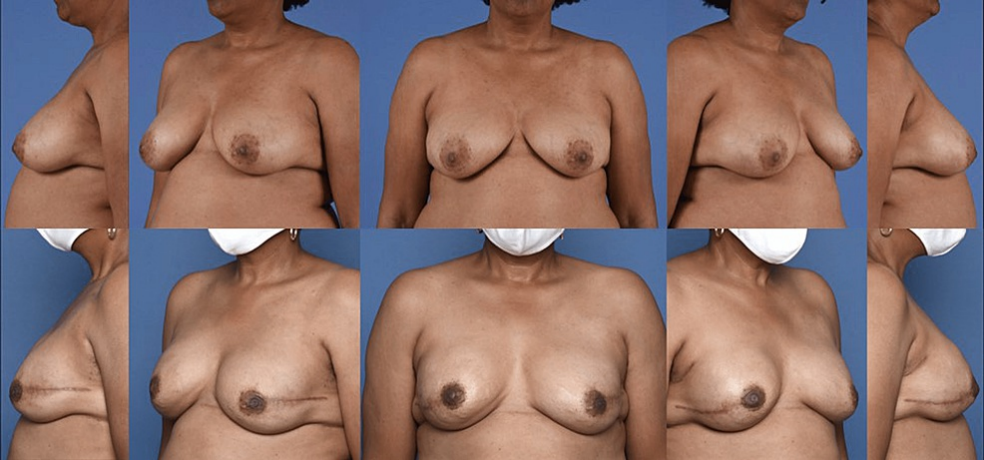
Preoperative and six-month postoperative comparison of non-ptotic breasts that underwent nipple-sparing mastectomy with staged-immediate, direct-to-implant, pre-pectoral implant-based breast reconstruction

Finally, the appropriate selection of patients for PP DTI breast reconstruction (ptotic and non-ptotic breasts) following NSM in the immediate or staged immediate timeframe requires an accurate assessment of breast characteristics and mastectomy skin/NAC perfusion following mastectomy (Figure 3).

In our series, seven patients initially planned for immediate reconstruction were modified to staged-immediate reconstruction timing based on intraoperative clinical findings and/or fluorescent angiography results. One of these patients experienced a complication following implant placement (cellulitis that resolved with IV antibiotics). In this subgroup of patients, no implants were lost and all achieved reconstruction completion, suggesting that the use of this reconstruction timing option, or a switch to it, may reduce implant risks while maintaining many of the benefits of immediate PP DTI reconstruction.

The data presented here represent a single surgeon's experience with SI DTI PP reconstruction after nipple-sparing mastectomy in patients with and without breast ptosis. This study has limitations, and the data must be interpreted in the proper context. Overall, the patient population analyzed was small, with the average patient being healthy and non-smoking, which may not represent the general population. Furthermore, it is derived from a single reconstructive surgeon, and three different surgical oncologists were responsible for the nipple-sparing mastectomies. Future directions of this study would also include a cost analysis of SI DTI PP reconstruction. This would also analyze the cost and complications of the procedure and compare it to traditional implant-based reconstructive techniques. Comparison studies with larger populations are warranted to advance this work.

## Conclusions

The utilization of SI timing allows for the majority of mastectomy-related problems to occur prior to the placement of implants, likely mitigating their effects on reconstruction following NSM, regardless of ptosis grade. Although a second procedure is needed for this reconstructive timing variation, the majority of the study population achieved reconstruction completion at the time of implant placement without further revision. The findings described in this article support the utility of SI timing in DTI PP breast reconstruction following NSM.
